# Antiproliferative Activity of *T. welwitschii* Extract on Jurkat T Cells *In Vitro*

**DOI:** 10.1155/2015/817624

**Published:** 2015-10-18

**Authors:** Batanai Moyo, Stanley Mukanganyama

**Affiliations:** Biomolecular Interactions Analyses Laboratory, Department of Biochemistry, University of Zimbabwe, P.O. Box MP 167, Mount Pleasant, Harare, Zimbabwe

## Abstract

*Triumfetta welwitschii* is a plant used traditionally for the treatment of fever and diarrhoea. Previous work has shown that *T. welwitschii* has antibacterial activity. The purpose of this study was to investigate *T. welwitschii* extract for anticancer activity against Jurkat T cells. The Jurkat T cell line is used to study acute T cell leukaemia. An antiproliferation assay, determination of induction of apoptosis, the determination of the effect of the combination of the extract and GSH, and effects of the extract on DNA leakage were conducted. *T. welwitschii* was found to decrease cell viability in a dose- and time-dependent manner. *T. welwitschii* caused apoptosis in the Jurkat T cells as shown by DNA fragmentation. When *T. welwitschii* was combined with reduced GSH, it was found that the growth of the Jurkat T cells was significantly reduced compared to untreated cells after 72 h of treatment. This was unexpected, as cancer cells have elevated levels of GSH compared to normal cells. The results of this study show that *T. welwitschii* is a potential source of compounds that may serve as leads for anticancer compounds.

## 1. Introduction

Cancer is a leading cause of death worldwide, accounting for 13% of all deaths worldwide in 2008 [1]. In developing countries, cancer is the third leading cause of death after infectious and cardiovascular diseases [2]. According to the World Health Organisation, there may be 21.4 million cases of cancer and 13.2 million deaths from cancer annually by 2030 [3]. Genetic and molecular modifications such as transformation, deregulation of apoptosis, proliferation, invasion angiogenesis, and metastasis are characteristics of cancer [4].

Leukaemia is a type of cancer where white blood cells and their precursors multiply and differentiate abnormally, resulting in a decrease in the production and function of normal cells [5]. Generally, chemotherapy, surgery, and radiation are the standard modes of treatment of cancer [3]; however, some of these procedures have been associated with side effects and drug resistance, particularly at high doses. Given that leukaemia is a systemic disease, its treatment and cure depends on chemotherapy rather than surgery [6]. Chemotherapy, radiation, immunotherapy, stem cell transplants, targeted therapy, and splenectomies are examples of standard leukaemia treatments [7].

The use of natural plant products such as plant extracts may reduce the side effects associated with cancer treatment [8]. The large number of components in the plants may overcome limitations of chemo- and targeted therapies, showing great anticancer potential [9]. A variety of compounds can be used as starting points for the synthesis of new drugs with improved activity [10]. Between 1983 and 1994, 60% of approved anticancer drugs were from natural sources [11]. A water extract of green tea leaves was recently approved for use as a drug by the Food and Drug Administration (FDA), proving that plant products can meet the high standards required by the FDA [4].

Plant compounds used in traditional medicine are generally considered safe as they are usually consumed as part of the diet [9]. An increase in the consumption of fruits, vegetables, whole grains, and spices has been seen to result in a decrease of the number of incidences of various types of cancer [5, 8, 9]. Plants contain natural compounds such as polyphenols that are known to reduce the risk of diseases such as cancer, diabetes, and neurodegenerative diseases [12].

Plant products, thus, play a large role in cancer prevention and, as documented in other studies, serve as a source of drugs [13]. Several studies conducted have shown that some plant extracts have anticancer activity [4, 14–18]. An example of a cancer drug from a plant is paclitaxel from* Taxus brevifolia*, which is also known as the northwest Pacific Yew tree [19]. Although most cancer drugs currently in use were isolated from plants, some of them have been shown to have side effects. The observed side effects are possibly due to the fact that single, pure compounds are used. A whole extract is expected to reduce or completely avoid the side effects.

*T. welwitschii* var.* welwitschii* is a shrubby plant that grows in Africa.* T. welwitschii* is used traditionally for the treatment of diarrhoea [20] and fever [21]. Fever is a symptom of inflammation, and chronic inflammation has been linked to cancer in some cases [22]. We have shown that* T. welwitschii* has antibacterial activity (unpublished results).

The aim of this study was to determine the effects of* T. welwitschii* var.* welwitschii* extract on Jurkat T cells. Jurkat T cells are a cell line used to study acute T cell leukaemia, T cell signalling [23], and the expression of various chemokines.

## 2. Materials and Methods

### 2.1. Chemicals

All chemicals, sera, media, and drugs used were purchased from Sigma-Aldrich (Steinheim, Germany) and were of analytical grade. These included foetal bovine serum (FBS), Roswell Park Memorial Institute 1640 media (RPMI), reduced L-glutathione (GSH), penicillin, neomycin, and streptomycin solution (PNS), Hanks Buffered Saline Solution (HBSS), methanol, dimethyl sulfoxide (DMSO), agarose, ethidium bromide (EtBr), Trypan blue dye, monochlorobimane (MCB), 2-4 dinitrochlorobenzene, and doxorubicin.

### 2.2. Plant Material

*Triumfetta welwitschii* var.* welwitschii* was collected from Centenary, Mashonaland Central, Zimbabwe (16.8°S, 31.1167°E, and 1 156 m above sea level), and identified by Mr. Christopher Chapano of the Harare Botanical Gardens, Zimbabwe. Dried roots were ground to a fine powder using a blender (Philips Co., Shanghai, China). Herbarium samples were stored in the Biomolecular Interactions Analysis Laboratory (BIA) Herbarium, University of Zimbabwe. Methanol was used for the extraction of compounds. To 20 g of powder, 200 mL of methanol was added. After 20 min, the plant extract was filtered using Whatmann number 1 filter paper and then dried under a stream of air until the methanol had evaporated. To prepare the extract solutions for testing, the plant extract was dissolved in DMSO and RPMI media, so that the final concentration of DMSO when added to test well was 0.83%. DMSO at a concentration of 0.83% was not found to be toxic to the Jurkat T cells.

### 2.3. Jurkat T Cells

Jurkat E6.1 human leukaemic T cell lymphoblasts (Jurkat T cells) were obtained from the European Collection of Cell Cultures (ECACC) (Salisbury, UK). They were grown in RPMI-1640 media supplemented with 10% FBS and 1% PNS solution at 37°C with 5% CO_2_ in an incubator (Shellab CO_2_ Series, Sheldon Mfg. Inc., Cornelius, USA).

### 2.4. Antiproliferation Assay

*T. welwitschii* root methanol extracts at 0, 31.25, 62.5, 125, and 250 *μ*g·mL^−1^ (final concentration in the wells) were used. Doxorubicin (10 *μ*g·mL^−1^) was used as the positive control. To each well in a 12-well plate, 100 *μ*L of the plant extract or doxorubicin was added. The number of cells added to each well was 1 × 10^5^ cells·mL^−1^. The volume was brought to 3 mL by adding RPMI. The plates were incubated at 37°C and 5% CO_2_ for 72 h (Shellab CO_2_ Series, Sheldon Mfg. Inc., Cornelius, USA). Every 24 h, 100 *μ*L of cells was collected to count the number of cells in each well. To the 100 *μ*L of cells, 50 *μ*L of 0.4% Trypan blue was added before counting the number of cells using a microscope. The dead cells were stained blue while the live cells were not stained.

### 2.5. Determination of Induction of Apoptosis

Jurkat T cells were treated with* T. welwitschii* extracts at 31.25, 62.5, 125, and 250 *μ*g·mL^−1^ for 72 h before being used for this experiment. Cells treated with doxorubicin at 10 *μ*g·mL^−1^ and the vehicle control (DMSO) only were used as the positive and negative controls, respectively. The cells were centrifuged at 12 000 rpm for 5 min in a KK Centrifuge (Gemmy Industrial Corp., Taiwan) and then washed with PBS (pH 7.2). After discarding the supernatant, 200 *μ*L of lysis solution (10 mM Tris (pH 7.4), 5 mM EDTA, 0.2% Triton X-100) and 10 *μ*L of 1 mg·mL^−1^ proteinase K were added. The cells were left in a 56°C water bath overnight (Shaker Bath SBS30, Stuart Scientific, UK). Eight microliters of RNAase (100 *μ*g·mL^−1^) was added to each tube, and then the incubation continued at 37°C for 1 hour. Twenty microliters of 1.5 M NaCl was added, and then the tubes were inverted several times before being centrifuged at 12 000 rpm for 15 min. The supernatant was added to clean eppendorf tubes. Ice cold isopropyl alcohol (2x the volume) was added. The tubes were inverted several times and left at −80°C for 1 h. The tubes were centrifuged at 12 000 rpm for 15 min in a microcentrifuge (Centrifuge 5415C, Eppendorf, Berlin, Germany), and the supernatant was then discarded. The isolated DNA was allowed to air-dry before being resuspended in TE buffer (10 mM Tris-HCl (pH 7.4) and 0.5 mM EDTA).

Gel electrophoresis was conducted using a BioRad electrophoresis unit (BioRad, Hercules, USA). A gel was prepared using 1 g agarose and 100 mL of TAE buffer. Ethidium bromide (0.5 *μ*g·mL^−1^) was added to the gel before pouring. Loading buffer (12.5 mg bromophenol blue and 2 g sucrose in 5 mL distilled water) was added to each sample and constituted 1/5 of the total volume. Ten microliters of sample was added to the wells except in lanes 1, 9, and 10 where 5 *μ*L was used. A Thermo Scientific GeneRuler 100 bp Plus DNA Ladder was also run. Five microliters of the ladder was loaded onto the gel. The gel was run at 110 V until the dye had travelled down 80% of the length of the gel. The gel was observed under UV light and photographed (MiniBIS BioImaging System, Dnr BioImaging Systems, Jerusalem, Israel).

### 2.6. Effect of Reduced Glutathione on the Action of* T. welwitschii*

Glutathione is involved in maintaining a redox balance in cells, thus protecting cells from damage due to oxidative stress [9]. Thus, the effect of reduced glutathione (GSH) on the activity of* T. welwitschii* root methanol extract was investigated. This assay was conducted to determine if the GSH antagonises or enhances the effect of the plant extract on the cancer cells. To a 12-well plate, the following samples were added in triplicate: cells and media; cells, media, and GSH; cells,* T. welwitschii* root methanol extract, and media; cells,* T. welwitschii* root methanol extract, GSH, and media. The concentration of GSH used was 25 *μ*g·mL^−1^ and that of the plant extract used was 19 *μ*g·mL^−1^ (the IC_50_). The plates were incubated at 37°C with 5% CO_2_ for 72 hours. The Trypan blue dye exclusion method was used to count the cells using a microscope.

### 2.7. Determination of the Effect of* T. welwitschii* and Doxorubicin on Jurkat T Cells Using the Propidium Iodide Assay

The effect of* T. welwitschii* and doxorubicin on Jurkat T cells was analysed using propidium iodide. The assay was conducted according to the method described by Kelter et al. [24] with some modifications. Propidium iodide is capable of binding to the DNA in nonviable cells after passing through damaged membranes [24]. Cells at a density of 1 × 10^4^ cells·mL^−1^ were added to a 96-well plate and incubated overnight at 37°C, 5% CO_2_. Control wells containing media only were also prepared. After the overnight incubation,* T. welwitschii* and doxorubicin were added to wells in triplicate. The concentrations ranged from 0 to 200 *μ*g·mL^−1^ for* T. welwitschii* and from 0 to 8 *μ*g·mL^−1^ for doxorubicin.* T. welwitschii* and doxorubicin were also added to wells containing media only. The plates were incubated for 4 days. The media were aspirated from the wells, and then 7 *μ*g·mL^−1^ propidium iodide was added to each well. Fluorescence was measured at an excitation wavelength of 544 nm and an emission wavelength of 612 nm using a *f*_max⁡_ microplate spectrofluorometer (Molecular Devices, Sunnyvale, USA). The plates were frozen to kill all of the cells and then thawed before fluorescence was measured as before. Growth stimulation/inhibition was calculated as T/C × 100%, where T and C are the fluorescence readings of the test and control samples, respectively. T/C > 125% indicated stimulation, while T/C < 30% indicated cytotoxicity.

### 2.8. Determination of the Effect of* T. welwitschii* on Drug Efflux in Jurkat T Cells

Drug efflux is a mechanism of drug resistance in cancer cells [25]. The purpose of this assay was to determine if* T. welwitschii* root methanol extract is capable of blocking the efflux pumps in Jurkat T cells. Cells at a density of 2 × 10^6^ cells·mL^−1^ were washed in Hank's buffered salt solution (HBSS) and centrifuged at 2000 rpm for 5 minutes. The pellet was resuspended in ice-cold HBSS containing 10 mM HEPES (pH 7.4) and centrifuged at 2000 rpm for 5 min. HBSS containing 5 *μ*M monochlorobimane (MCB) was added to the cells, which were then incubated for 1 hr in a waterbath at 10°C. The cells were washed twice in HBSS containing 11.1 mM glucose. After washing, the cells, resuspended in HBSS with glucose, were divided equally between six tubes, each with an equal concentration of cells. Two tubes served as an untreated control. To two tubes* T. welwitschii* was added at a concentration of 10 *μ*g·mL^−1^ (enough to have an effect but not to kill the cells), and to the last two tubes, CDNB was used as the positive control at 1 mM. CDNB reacts with glutathione to form 2,4-dinitrophenylglutathione. This product is pumped out of the cell via efflux pumping and therefore will complete with glutathione-bimane [26]. The samples were incubated for 1 hr at 37°C and then centrifuged at 2000 rpm for 5 min. The supernatant was collected. The fluorescence of the plates was measured at 390 nm and 510 nm (excitation and emission, resp.) using a *f*_max⁡_ microplate spectrofluorometer (Molecular Devices, Sunnyvale, USA).

### 2.9. Statistical Analysis

GraphPad Prism 5 for Windows (GraphPad Software Inc., San Diego, California, USA) version 5.03 was used to analyse the results using one-way analysis of variance test (ANOVA) with Dunnett's Multiple Comparison Test. Values with a *P* value 0.05 or less were considered statistically significant.

## 3. Results

### 3.1. Antiproliferation Assay

**Figure 1:**
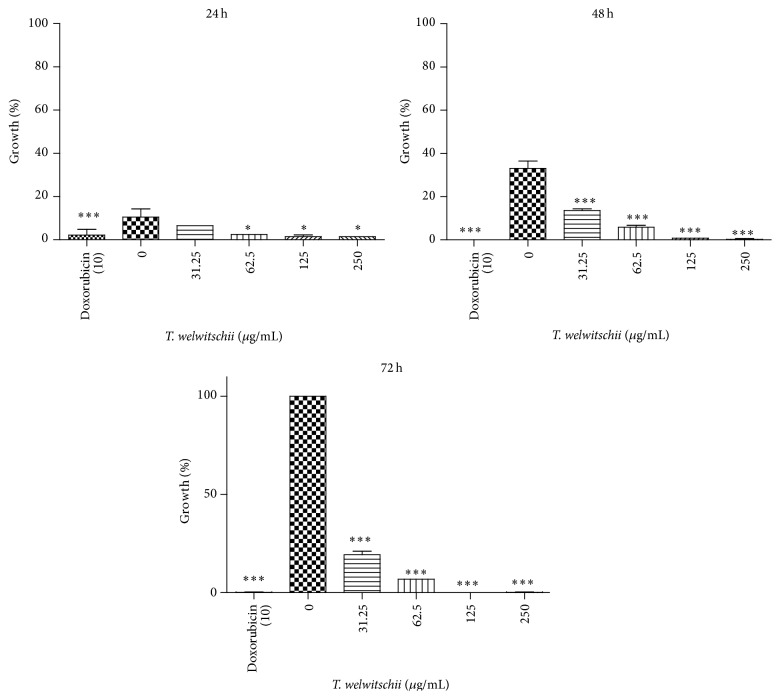
Percentage surviving cells after exposure to* T. welwitschii* and doxorubicin for 24, 48, and 72 h. The number of live negative control cells (0 *μ*g·mL^−1^) at 72 h was used as 100%, so all cells were expressed as a percentage of them. Values indicate the mean ± SD for *n* = 3. ^*∗*^ *P* < 0.05, ^*∗∗*^ *P* < 0.01, and ^*∗∗∗*^ *P* < 0.001.

The results of the antiproliferation assay are shown in Figure 1. The most rapid growth of the untreated cells was observed between 48 h and 72 h, where the cell viability increased from 33.2% to 100%. As the concentration of the plant extract increased, the percentage of live cells decreased. Cells treated with 250 *μ*g·mL^−1^ *T. welwitschii* had viabilities of less than 0.5%. Cells treated with doxorubicin did not survive treatment. When the cells were treated with 31.25 *μ*g·mL^−1^ and 62.5 *μ*g·mL^−1^ of the extract, the percentage of live cells increased over 72 h but did not rise above 19.38% (31.25 *μ*g·mL^−1^). At 125 *μ*g·mL^−1^ and 250 *μ*g·mL^−1^ of plant extract, the cell viability decreased with time. Doxorubicin was used at s concentration of 10 *μ*g·mL^−1^ and was found to have growth inhibition activity against the Jurkat T cells. At 24 h, cell viability was 2.1% but decreased to 0.1% at 72 h.

### 3.2. Determination of Induction of Apoptosis

**Figure 2:**
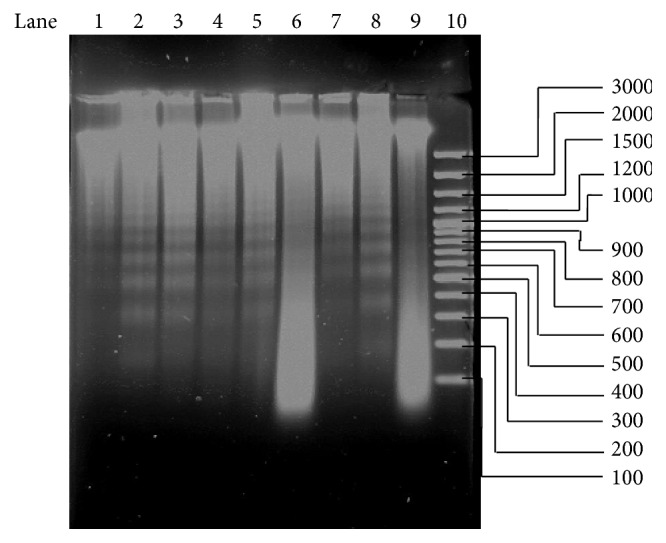
Gel electrophoresis following treatment with* T. welwitschii* extracts at various concentrations. For lanes 2–8, 10 *μ*L was loaded, while for the rest, 5 *μ*L was loaded. The results of the assay show that DNA fragmentation was caused by the Jurkat T cells. Lanes 1, 7: 10 *μ*g·mL^−1^ doxorubicin (5 *μ*L in lane 1, 10 *μ*L in lane 7). Lanes 2, 3, 4, 5, and 6:* T. welwitschii* 250, 125, 62.5, and 31.25 *μ*g·mL^−1^. Lane 8:* T. welwitschii* 250 *μ*g·mL^−1^. Lane 6, 9: 0 *μ*g·mL^−1^ (5 *μ*L). Lane 10: GeneRuler 100 bp Plus DNA Ladder (5 *μ*L).

The results of this assay are shown in Figure 2. The photograph of the gel shows DNA laddering caused by doxorubicin and* T. welwitschii* at various concentrations. A 1 kb pair marker was run in lane 10 to measure the bands. A large band signifying intact DNA was observed in the lanes containing untreated cells (lanes 6 and 9). More DNA fragmentation was observed in the cells treated with* T. welwitschii* than those treated with doxorubicin. The sizes of the bands ranged from larger than 3000 bp to smaller than 100 bp. Small (<100 bp) fragments were observed in the untreated cells. Some DNA in all of the lanes except the one with the marker failed to migrate from the wells. The bands appeared as smears as they tended to run into each other.

### 3.3. Effect of Reduced Glutathione on the Activity of* T. welwitschii*

**Figure 3:**
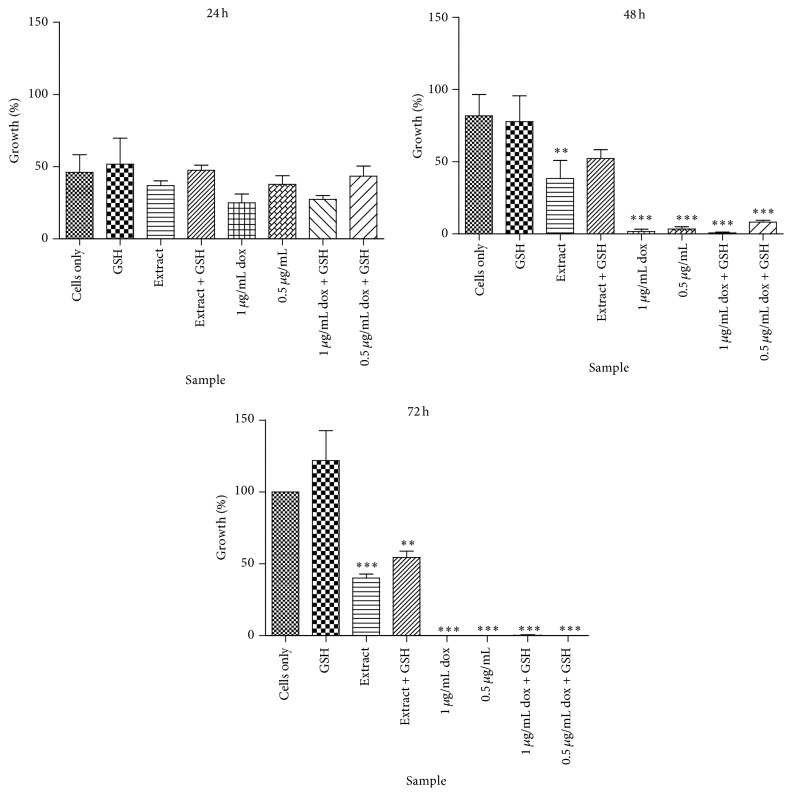
The effects of glutathione on the activity of* T. welwitschii*. Glutathione (GSH) was used at a concentration of 25 *μ*g·mL^−1^, while a concentration of 19 *μ*g·mL^−1^ of* T. welwitschii* root methanol extract (extract) was used in this assay. Doxorubicin (dox) was tested against the Jurkat T cells at 1 *μ*g·mL^−1^ and 0.5 *μ*g·mL^−1^. The cells were incubated over a period of 72 h, and cell counts conducted every 24 h using the Trypan blue exclusion assay. To determine significance, all treatments were compared to cells only. Values indicate the mean ± SD for *n* = 3. ^*∗∗*^ *P* < 0.01, ^*∗∗∗*^ *P* < 0.001.

Figure 3 shows the effect of GSH on the activity of* T. welwitschii* root methanol extract against Jurkat T cells. The extract showed dose-dependent activity against the Jurkat T cells. The growth of cells was 36.9% at 24 h, 38.3% at 48 h, and 40% at 72 h. GSH did not cause any significant changes in the growth of cells at 72 h. Combining the extract with GSH resulted in increased growth of the Jurkat T cells compared to the extract alone. When GSH was combined with doxorubicin, cell growth increased slightly at 24 h and 48 h compared to cells treated with doxorubicin only, while at 72 h there were no live cells.

### 3.4. Effect of* T. welwitschii* on Drug Efflux in Jurkat T Cells

**Figure 4:**
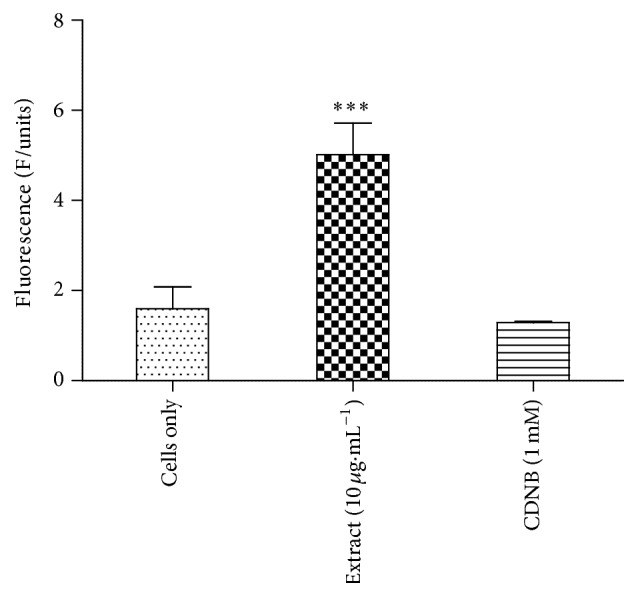
The effects the plant extract on the efflux of the MCB-GSH conjugate from Jurkat T cells. The graph shows the effect of* T. welwitschii* root methanol extract at a concentration of 10 *μ*g·mL^−1^ on the efflux of the MCB-GSH conjugate. CDNB was used as the positive control while the negative control, cells only, was treated with the vehicle control. Values indicate the mean ± SD for *n* = 8. ^*∗∗∗*^ *P* < 0.001.

The results of this assay are shown in Figure 4. In the control group, there was slightly more efflux than accumulation (1.6 fluorescence units (F/units) and 1.5 F/units, resp.). Treating the Jurkat T cells with the extract increased efflux of the monochlorobimane-glutathione (MCB-GSH) conjugate from the cancer cells compared to accumulation (5 F/units and 1.6 F/units, resp.). CDNB which is converted to CDNB-GSH caused slightly more accumulation of the MCB-GSH conjugate in the Jurkat T cells than efflux (1.5 F/units and 1.3 F/units, resp.).* T. welwitschii* extract enhanced the efflux of the MCB-GSH conjugate from the Jurkat T cells.

### 3.5. The Effect of* T. welwitschii* and Doxorubicin on Jurkat T Cells Using Propidium Iodide

**Figure 5:**
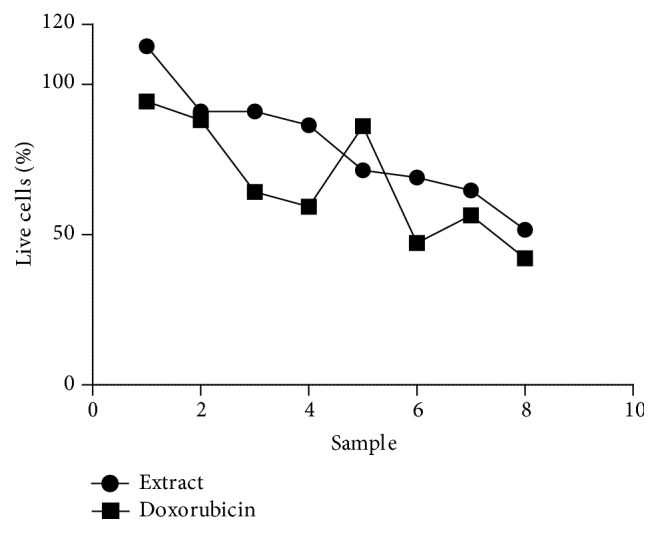
Effect of* T. welwitschii* and doxorubicin on Jurkat T cells measured using the propidium iodide assay. Jurkat T cells were exposed to the plant extract and doxorubicin alone and in combination before the effect was measured using propidium iodide at excitation and emission wavelengths 544 nm and 612 nm, respectively. The growth of the cells is expressed as test/control × 100%.* T. welwitschii*: 1: 3.125 *μ*g·mL^−1^; 2: 6.250 *μ*g·mL^−1^; 3: 12.50 *μ*g·mL^−1^; 4: 25 *μ*g·mL^−1^; 5: 50 *μ*g·mL^−1^; 6: 100 *μ*g·mL^−1^; 7: 150 *μ*g·mL^−1^; 8: 200 *μ*g·mL^−1^. Doxorubicin: 1: 0.25 *μ*g·mL^−1^; 2: 0.5 *μ*g·mL^−1^; 3: 1 *μ*g·mL^−1^; 4: 2 *μ*g·mL^−1^; 5: 3 *μ*g·mL^−1^; 6: 4 *μ*g·mL^−1^; 7: 5 *μ*g·mL^−1^; 8: 8 *μ*g·mL^−1^.

The purpose of the propidium iodide assay was to determine the effect of* T. welwitschii* extract and doxorubicin on Jurkat T cells using propidium iodide. Propidium iodide is only capable of entering cells with damaged membranes and binding to their DNA, thus making it an excellent tool for measuring cell viability [24]. The results of this assay are shown in Figure 5. It was found that increasing the concentration of* welwitschii* resulted in decreased levels of percentages of live cells. At 3.125 *μ*g·mL^−1^ *T. welwitschii* the percentage of live cells was 113%. This decreased to 52% at 200 *μ*g·mL^−1^. There was no difference between 6.25 *μ*g·mL^−1^ and 12.5 *μ*g·mL^−1^, in the percentage of live treated cells. At 50 *μ*g·mL^−1^, 100 *μ*g·mL^−1^, and 150 *μ*g·mL^−1^ of* T. welwitschii*, the percentages of live treated cells were similar. Increasing the concentration of doxorubicin decreased the percentage of live cells. The range of percentages of live cells treated with doxorubicin was generally lower than the range for the cells treated with the extract.

## 4. Discussion

Cancer has become one of the top killer diseases worldwide with the numbers of cases and deaths being expected to increase over the next 15 years [3]. An increase in the incidences of drug resistant cancer and terrible side effects has resulted in a need for new anticancer compounds with diverse modes of action and little to no side effects. As whole extracts contain a variety of compounds, they will most likely have a variety of targets. Plants have been used for centuries to treat a variety of illnesses. It has been reported that eating fruit and drinking fruit juices during the first two years of life resulted in a decrease in the incidences of leukaemia in children under the age of 15 years [27].

In this study,* Triumfetta welwitschii* root methanol extract was tested against Jurkat T cells.* T. welwitschii* inhibited the growth of Jurkat T cells in a dose-dependent manner. As the concentration of the plant extract increased, the number of live cells decreased. The action of 10 *μ*g·mL^−1^ doxorubicin was comparable to that of 250 *μ*g·mL^−1^ of the plant extract. At 31.25 *μ*g·mL^−1^ *T. welwitschii*, the number of live cells had decreased tremendously compared to the untreated cells, showing that large concentrations of the extract were not needed to inhibit the growth of the cells. The plant extract was very effective against Jurkat T cells. Other studies have found plant extracts to have antiproliferative activity against Jurkat T cells, for example,* Hemidesmus indicus* at concentrations ranging from 0.62 mg·mL^−1^ to 1.9 mg·mL^−1^ [4].* Triumfetta rhomboidea* has antitumour activity* in vivo* [28, 29].* Grewia hirsuta*, a Tiliaceae plant, has been found to have anticancer activity against HepG2 cells by arresting the cell cycle [29].

The low percentage of live cells observed in the treated cells show that the plant extract had effective antiproliferative activity against the Jurkat T cells. The concentration needed to reduce the growth of the Jurkat T cells to 50% was 19 *μ*g·mL^−1^. This concentration is considered to be very low, as, according to the American National Cancer Institute, a value of 30 *μ*g·mL^−1^ is the highest IC_50_ that is considered promising when searching for activity in whole extracts [11]. The extract may contain phytochemicals that have potential anticancer properties. When testing the effects of any new compound, on cancer cells, cells are exposed to the compound or extract whilst control cells are not exposed the test compound. Thus, the data produced in our study points to the effects of the extract on exposed compared to cells that were not exposed to the extract. In this regard, our data does is not able elucidate whether the action of the extract was specific or nonspecific. There is, therefore, a need for further studies to be done on normal cells or control cells such as PBMCs.

The clumping of the cells observed* in vitro* could be a result of toxicity as has been observed in other studies with plant extracts [30]. According to Chung et al., leukaemia cells tend to adhere to each other* in vitro* either spontaneously or in response to mitogens and some drugs [30]. A surface molecule, *β*-catenin, regulates cell-cell adhesion of leukaemic cells [30].

Apoptosis is used by the body to regulate the proliferation of cells [31]. Consequently, an imbalance or inactivation of pathways that regulate apoptosis can result in the formation of tumours and cancer progression [31]. As DNA cleavage is a sign of apoptosis, the DNA laddering assay was conducted. Exposure to the plant extract caused more DNA damage than doxorubicin, which is known to induce apoptosis. In lanes 6 and 9, the DNA from the untreated cells was added. Large bands of DNA that were smaller than 300 bp were observed indicating intact DNA fragments. The banding pattern between the treated and untreated cells can, however, be distinguished as the treated cells show DNA laddering compared to the large intact band seen with the untreated cells. Previous studies found plant extracts that caused DNA fragmentation in Jurkat T cells, for example, fermented wheat germ extract [32] and* Ziziphus jujuba* [33].

GSH is involved in the maintenance of a balanced redox environment within cells [34]. In this study, the effects of GSH on the action of* T. welwitschii* root methanol extract were investigated. When the cells were treated with GSH and the plant extract, the growth of the Jurkat T cells was significantly lower than the untreated cells after 72 h. Compared to the extract alone, when GSH and the plant extract were combined, there was an increase in the number of live cells, but this was still lower than the untreated control. GSH would antagonise the action of the plant extract if the extract acted via a redox mechanism [26].* T. welwitschii* may, therefore, not act on the Jurkat T cells via a redox pathway. The plant extract may act through the inhibition of a particular enzyme, physiochemical mechanisms, by targeting the DNA/RNA or by targeting ion channels [35].

When the cells were exposed to GSH alone, no significant increase in cell growth was observed. In other studies, it has been observed that some increase in cell growth was expected as GSH protects against reactive oxygen species, mutagens, and drugs [36]. GSH also plays a role in multidrug resistance by reacting with drugs spontaneously [36]. Tumour cells have more intracellular GSH than normal cells [37] and the depletion of GSH makes cancer cells more susceptible to reactive oxygen species (ROS) and anticancer agents [36]. GSH has been found to induce cell death in HL-60 leukaemia cells in concentrations ranging from 20 *μ*g·mL^−1^ to 2 000 *μ*g·mL^−1^ [38]. Further work needs to be done to determine the mode of action of the* T. welwitschii* extract when combined with GSH. Methanol extracts contain many phytoconstituents, including anthocyanins, saponins, xanthoxyllines, lactones, flavones, polyphenols, and tannins [39]. Some of these phytochemicals may form conjugates with glutathione that can potentially inhibit the glutathione-dependent enzymes [26].

Propidium iodide is a fluorescent dye that cannot penetrate live cells and binds to DNA [40]. An increase in the fluorescence of propidium iodide corresponds to an increase in the number of dead cells [24]. The results of the study conducted showed that, on their own,* T. welwitschii* and doxorubicin were not cytotoxic towards the Jurkat T cells as, at all tested concentrations, T/C × 10% is greater than 50%. However, a decrease in the percentage of live treated cells was observed as the concentrations of doxorubicin and* T. welwitschii* increased. The doxorubicin was more effective against the cells as the range of percentages of live treated cells was lower even though the concentrations used were much lower. The results of the antiproliferative assay showed the extract and doxorubicin were cytotoxic against the Jurkat T cells. Although the extract is toxic against the cells, the extract may not damage the integrity of the cell membrane. Propidium iodide is only capable of staining nucleic acids in cells with damaged membranes [24]. Apoptosis may account for the differences in the results observed between the two assays. Following apoptosis, complete degradation of the nucleic acids may have occurred resulting in no positive results, as there would be no nucleic acids for the propidium iodide to bind to. The Trypan blue dye exclusion counting method may, therefore, be considered more accurate than the propidium iodide assay. The accuracy of the counting method is increased by the ability to visualize live and dead cells. Whilst our results are not conclusive of the apoptotic process in themselves, the presence of DNA ladders and the staining of cells by propidium iodide in cell exposed to the extract as compared to the control give indications to possible mechanism of actions. Propidium iodide is capable of binding to the DNA in nonviable cells after passing through damaged membranes. The loss of membrane integrity, thus, could be ascribed to the exposure to the extract as this was not observed in the control cells. After isolation of active phytoconstituents from the crude extract, more comprehensive apoptotic assays would be required to elucidate the mode of cell death.

Chemotherapy can be rendered ineffective by the development of drug resistance. Decreased drug accumulation within a cell is one of several molecular mechanisms proposed to explain drug resistance [25]. A reduction in drug influx via drug solute carriers or increased drug efflux via ATP-binding cassette efflux pumps contributes to a reduction in drug accumulation [25]. The effect of* T. welwitschii* on drug transport in Jurkat T cells was investigated. In this assay, CDNB was used as the positive control as CDNB via its glutathione conjugate is an efflux pump inhibitor [26]. It was found that* T. welwitschii* root methanol extract at 10 *μ*g·mL^−1^ stimulated efflux of the MCB-GSH conjugate from the Jurkat T cells. The fluorescence of effluxed MCB-GSH conjugate was much higher than the accumulated one, showing that the plant extract induces the efflux of large amounts of the conjugate. A low concentration of the extract was used to prevent cell death. As the plant concentration increased there was corresponding cytotoxicity observed and, therefore, further studies are needed to determine if the mode of action at higher concentrations could be through inhibition of drug efflux.* Cecropia lyratiloba*,* Panax* spp.,* Euphorbia serrulata*, and* Ruta graveolens* are examples of plants that contain compounds with drug efflux inhibitory activity [41].

In summary, the results of this study showed that* T. welwitschii* has antiproliferative activity against Jurkat T cells. The effects of the extract were also shown to be irreversible as the plant extract induced DNA fragmentation.* T. welwitschii* is a potential source of lead compounds for new antileukaemic medicines. Key et al. [42] noted that some nonnutrient chemicals in plants have been found to have anticancer activity* in vitro*. Some of the classes are carotenoids, sulphur-containing compounds, and phytosterols [42]. Several medicinal plants have been found to be toxic to humans, for example,* Ziziphus mucronata* and* Athrixia phylicoides* [43]. Examples of side effects include diarrhoea, immunotoxicity, embryo/foetal and prenatal toxicity, cardiovascular side effects, and hypersensitivity [43]. At times the toxic effects are only seen at high doses, so low doses are required. For that reason, the testing of* T. welwitschii* root methanol extract against normal human cell lines such as peripheral blood mononuclear cells (PBMCs) and normal mouse fibroblast NIH/373 cells is important in order to determine if the extract is toxic against them to ensure that only leukaemic cells are targeted by the extract. The viability of the PBMCs and NIH/373 cells after exposure to the extract will be determined in future studies.

## 5. Conclusion

The* Triumfetta welwitschii* extract was shown to have antiproliferative activity against Jurkat T cells and its effects were shown to be irreversible.* T. welwitschii* plant may, thus, serve as a potential source of lead compounds for anticancer compounds.
